# Polymer Nanocomposites Based on Nanosized Substituted Ferrites (NiZn)_1−x_Mn_x_Fe_2_O_4_ on the Surface of Carbon Nanotubes for Effective Interaction with High-Frequency EM Radiation

**DOI:** 10.3390/ma17050986

**Published:** 2024-02-21

**Authors:** Ruslana Mazurenko, Serhii Prokopenko, Marcin Godzierz, Anna Hercog, Anastasiia Kobyliukh, Grygorii Gunja, Stanislav Makhno, Urszula Szeluga, Petro Gorbyk, Barbara Trzebicka

**Affiliations:** 1 Chuiko Institute of Surface Chemistry, NAS of Ukraine 17 General Naumov Str., 03164 Kyiv, Ukraine; sprokopwork@gmail.com (S.P.); g.m.gunya2020@gmail.com (G.G.); stmax@ukr.net (S.M.); phorbyk@ukr.net (P.G.); 2 Centre of Polymer and Carbon Materials, Polish Academy of Sciences, 34 M.C. Sklodowska Str., 41-800 Zabrze, Poland; ahercog@cmpw-pan.pl (A.H.); akobyliukh@cmpw-pan.pl (A.K.); uszeluga@cmpw-pan.pl (U.S.); btrzebicka@cmpw-pan.pl (B.T.); 3 Faculty of Chemistry, Ningbo University of Technology, 201 Fenghua Road, Ningbo 315211, China

**Keywords:** nanosized spinel ferrites, carbon nanotubes, polymer matrix systems, absorption coefficient, reflection loss

## Abstract

To create materials that interact effectively with electromagnetic (EM) radiation, new nanosized substituted ferrites (NiZn)_1−x_Mn_x_Fe_2_O_4_ (x = 0, 0.5, and 1) anchored on the surface of multi-walled carbon nanotubes (CNTs) have been synthesized. The concentration of CNTs in the (NiZn)_1−x_Mn_x_Fe_2_O_4_/CNT system was from 0.05 to 0.07 vol. fractions. The dielectric and magnetic characteristics of both pristine (NiZn)_1−x_Mn_x_Fe_2_O_4_ ferrites and (NiZn)_1−x_Mn_x_Fe_2_O_4_/CNT composite systems were studied. The introduction of (NiZn)_1−x_Mn_x_Fe_2_O_4_/CNT composites into the amorphous epoxy matrix allows to tailor absorbing properties at the high-frequency by effectively shifting the maximum peak values of the absorption and reflection coefficient to a region of lower frequencies (20–30 GHz). The microwave adsorption properties of (NiZn)_1−x_MnxFe_2_O_4_/0.07CNT–ER (x = 0.5) systems showed that the maximum absorption bandwidth with reflection loss below −10 dB is about 11 GHz.

## 1. Introduction

The intensive development of radio and electronic technology, encompassing wireless and satellite communications, navigation and radar systems, radio installations, medical radio equipment, portable computers, and more, has resulted in the emergence of additional electromagnetic pollutions [1]. Most of these technical systems utilize microwave energy. The impact of electromagnetic radiation (EMR) within the microwave range (1–40 GHz) on technical and biological entities stands as a crucial factor influencing their functions and activities [2]. Hence, the exploration of new nanomaterials with enhanced efficiency in absorbing electromagnetic interference is of significant interest to both the academic and industrial communities.

In contemporary research on electromagnetic wave-absorbing materials, various substances are employed for their ability to absorb electromagnetic radiation within specific frequency ranges. Numerous studies and review articles now delve into different approaches to creating EM interference shielding materials. These approaches encompass various ferrites (differing in shapes and sizes) and substituted ferrites, ceramic materials, bimetals, nanocomposites featuring conductive and magnetic components, nanocomposites with diverse conductive polymer matrices, and core/shell nanocomposites [3,4,5,6,7].

The capacity of a material to absorb electromagnetic radiation hinges on its electrical and magnetic properties, which encompass electrical conductivity and, dielectric and magnetic permeability. These characteristics play a pivotal role in analyzing the process of EM wave propagation, and they are typically nonlinear, tensor, and complex quantities. When EMR interacts with an absorbing material, processes such as reflection, absorption, multiple scattering (due to the structural and geometric inhomogeneity of the material), and wave interference come into play. The absorption of electromagnetic energy occurs due to dielectric, magnetic, and conduction losses, which tend to be maximized to achieve optimal shielding efficiency. Simultaneously, when electromagnetic waves strike a material, reflection occurs at the interface between the media. A greater discrepancy between the wave impedances of the media results in a higher reflection coefficient [8].

The primary objective in the development of absorbing materials is to align the absorbing structure with the surrounding space, minimizing the integral effect of reflection. Considering the operating principles, absorbent materials can be categorized into interference (utilizing the principle of mutual damping of electromagnetic waves by superimposing incident and reflected waves in antiphase), dissipating (reducing reflected energy by scattering it in other directions at various angles), absorbing (transforming an electromagnetic wave into other types of energy, typically thermal, due to dielectric and magnetic material losses), and combined (combining different principles of action) [9].

Maintaining a balance between the dielectric and magnetic characteristics of nano-materials is crucial for the efficient absorption of electromagnetic waves that penetrate the material deeply. This balance is essential since absorption is facilitated by the interaction of electric and magnetic dipoles with external electromagnetic fields, matching with the impedance of the environment. Thus, to enhance the absorption loss of interfering electromagnetic waves, absorbent materials must exhibit appropriate conductivity, dielectric permittivity, and permeability. Nowadays polymer composite materials based on various polymer matrices filled with carbon materials (carbon nanotubes, graphene nanoplatelets, carbon fibers, graphite) are actively investigated and gain considerable interest as electromagnetic interference (EMI) shielding materials [8,10,11,12,13,14]. Electron conducting materials can serve as a shield to minimise or even eliminate interference phenomena by primarily reflecting electromagnetic waves. By introducing conductive fillers inside the polymer matrix, a lightweight conductive material with electromagnetic interference shielding properties can be obtained [10,11]. The obtained epoxy composite materials demonstrated high shielding reflection of EM waves of microwave range. However, in order to obtain the materials with high absorption it is necessary to reduce the electrical conductivity of the filler and thus reduce the reflection of the electromagnetic wave.

The most promising absorbent materials, according to technological, operational, and economic criteria, are those originating from a component exhibiting electrical conductivity (nanocarbon materials) that are enhanced by a magnetic component (spinel ferrites). Nanosized spinel ferrites (MFe_2_O_4_, where M = Co^2+^, Ni^2+^, Zn^2+^, etc.) are effective in EMR absorption applications as a consequence of their elevated saturation magnetization and magnetic losses, which include hysteresis loss and eddy current loss [15]. Ferrites exhibit several dielectric and magnetic resonances that beneficially impact EMR absorption. The incorporation of carbon materials (such as graphene nanoplatelets, graphene oxide, and carbon nanotubes), owing to their excellent properties (i.e., being lightweight, having high corrosion resistance, and superior electrical and thermal properties) allows for the development of materials with tuneable electric/dielectric properties for EM shielding absorption [16]. However, these materials often exhibit strong surface reflection, inevitably causing the secondary reflection of EM waves. In spite of the effective composition of components, providing the material with the necessary higher electromagnetic interference (EMI) absorption losses, reflection losses concurrently increase.

Our preliminary studies have indicate that the development of nanocomposites, incorporating a conductive element modified by a magnetic component, demonstrates enhanced efficiency in absorbing electromagnetic waves within the microwave spectrum. This heightened effectiveness is particularly noticeable at optimal ratios of permittivity and permeability, outperforming components solely relying on pristine magnetic ferrite. [17]. Substituted ferrites enable the control of both magnetic permeability and magnetic losses in the microwave region. Therefore, the use of substituted ferrites (NiZn)_1−x_Mn_x_Fe_2_O_4_ is relevant for creating EM-absorbing materials [18,19]. Additional mechanisms for dissipation of electromagnetic energy in the microwave range with polymer-conductor systems have also been shown to be connected with polarization effects arising due to interphase interaction of the components [20,21].

It is well-known that the creation of polymer composites allows for improved manufacturability, enhanced performance parameters, and, superior functionality for such materials. Epoxy resins (ER) are widely used as polymer matrices due to their excellent mechanical properties, chemical and heat stability, antibacterial properties, strong adherence, and low contractibility [22,23]. Meanwhile, the introduction of fillers in the epoxy resin that interact with microwave electromagnetic radiation can improve the impedance matching between polymer composites and the environment and cause greater polarization of the interface in the composites [24,25].

The goal of this research was to create new potential nano-sized fillers based on substituted (NiZn)_1−x_Mn_x_Fe_2_O_4_ ferrites anchored on the surface of multi-walled carbon nanotubes that would be dedicated as active fillers of the epoxy matrix interacting with elec-tromagnetic radiation in the microwave ranges.

## 2. Materials and Methods

### 2.1. Materials

Iron (III) nitrate nonahydrate (Fe(NO_3_)_3_·9H_2_O, pure grade), manganese (II) nitrate hexahydrate (Mn(NO_3_)_2_·6H_2_O, pure grade), nickel (II) nitrate hexahydrate (Ni(NO_3_)_2_·6H_2_O, pure grade), and zinc nitrate hexahydrate (Zn(NO_3_)_2_·6H_2_O, pure grade) were procured from Chempur Co. (Piekary Slaskie, Poland). Sigma-Aldrich (Saint Louis, MO, USA) provided hydrazine monohydrate (64–65% N_2_H_4_, reagent grade 98%). Multi-walled carbon nanotubes (C ≥ 95%, average length 5 μm, average diameter 6–9 nm) were sourced from Sigma-Aldrich. Carl Roth GmbH Co. (Karlsruhe, Germany) produced Polyvinylpyrrolidone (K–90), characterized by a molar mass of 900,000–1,200,000 g/mol, a density of 1.2 g/cm^3^, and a melting point of 130 °C. The epoxy resin used was diglycidyl ether of bisphenol A (Epidian 6) with an epoxy group content of 0.54 mol/100 g, obtained from the chemical plant CIECH-Sarzyna (Nowa Sarzyna, Poland). This resin underwent cross-linking using tetra-methylenediamine (TETA, CIECH-Sarzyna, Nowa Sarzyna, Poland). All chemical reagents were of pure grade and were utilized without any purification.

### 2.2. Synthesis of (NiZn)_1−x_Mn_x_Fe_2_O_4_/CNT Powders

Carbon nanotubes underwent modification with spinel ferrites (NiZn)_1−x_Mn_x_Fe_2_O_4_ (x = 0, 0.5, and 1) using the co-precipitation method in the presence of hydrazine hydrate as a reducing agent. The concentration of CNTs in the (NiZn)_1−x_Mn_x_Fe_2_O_4_/CNT system ranged from 0.05 to 0.07 vol. fractions (ϕ). To achieve this, the requisite amount of CNTs was dispersed in deionized (DI) water using an ultrasonic disperser (Bandelin Sonopuls, Berlin, Germany) for 40 min. Stoichiometric amounts of Fe(NO_3_)_3_·9H_2_O, Ni(NO_3_)_2_·6H_2_O, Zn(NO_3_)_2_·6H_2_O, and Mn(NO_3_)_2_·6H_2_O were dissolved in DI water, followed by the addition of an appropriate amount of polyvinylpyrrolidone solution with continuous stirring for 40 min. Subsequently, the mixture was heated to 80 °C. The water dispersion of carbon nanotubes was introduced to the heated mixture with continuous stirring. Following this, an appropriate quantity of hydrazine hydrate was added until the pH was adjusted to 8.0, and the final mixture was boiled for 4 h. The powder was separated by magnetic separation and then dried at 80 °C for 7 h.

### 2.3. Fabrication of a Polymer-Based System Filled with Ferrite/CNTs Particles

Polymer-filled systems were formulated using an epoxy resin cross-linked with aliphatic amine and (NiZn)_1−x_Mn_x_Fe_2_O_4_/CNT powders. The necessary quantities of modified powders were incorporated into Epidian 6 and dispersed through mechanical stirring until a homogeneous mixture was achieved. To complete the polymer systems, triethylenetetramine was added in a stoichiometric proportion to the epoxy resin. The resulting blends were poured into silicone molds and initially cured at room temperature, followed by heat treatment at 80 °C for 2 h and an additional 2 h at 120 °C.

### 2.4. Electrophysical Measurements

The examination of the structures of both pristine ferrites and ferrites deposited onto carbon nanotube surfaces was conducted through transmission electron microscopy (TEM) on a Tecnai F20 TWIN microscope (FEI Company, Hillsboro, OR, USA) equipped with a field emission gun, operating at an accelerating voltage of 200 kV. For this analysis, a droplet (6.0 μL) of the dispersion of powdered samples in acetone was applied to a grid (Lacey Carbon Film, 200 mesh copper) and air-dried for 24 h. TEM microphotographs were captured using a Gatan Rio 16 CMOS 4k camera (Gatan Inc., Pleasanton, CA, USA) and processed with Gatan Microscopy Suite (GMS) software (Version 3.31.2360.0, Gatan Inc.).

The crystalline structures of synthesized samples, i.e., pristine ferrites and CNTs covered with ferrites, were determined by the D8 Advance diffractometer (Bruker AXS, Karlsruhe, Germany) with a Cu-K_α_ cathode (λ = 1.54 Å). The diffractometer operated at a voltage of 40 kV and a current of 40 mA, employing a Bragg-Brentano geometry. X-ray scattering curves were recorded over a diffraction angle (2θ) range of 2–50° with a step size of 0.02°, utilizing a LYNXEYE XE-T detector (Bruker AXS, Karlsruhe, Germany). Crystallite size calculations were performed through Rietveld refinement in the TOPAS 6 program, based on Williamson-Hall theory [26,27]. The pseudo-Voigt function was utilized in describing diffraction line profiles during the Rietveld refinement. Numerical criteria such as R_wp_ (weighted-pattern factor) and GOF (goodness-of-fit) parameters were employed to assess the quality of the fit relative to experimental diffraction data [28]. The dislocation density was computed using Equation (1), where *δ* represents dislocation density and *D* is crystallite size. The chemical composition of the as-synthesized ferrites and ferrite/CNT hybrids was determined using S2 Puma X-ray fluorescence spectroscopy (Bruker AXS, Karlsruhe, Germany).

(1) $$\delta =\frac{1}{D^{2}}$$

The complex dielectric permittivity, with its real (ε′) and imaginary (ε″) components, and the complex magnetic permeability, with its real (μ′) and imaginary (μ″) components, for composites at microwave frequencies (9 GHz) were determined using an interferometer (RFK 2-18, measuring phase differences) and a standing wave meter (R2-60) through an electrodeless method [29]. The testing involved polymer-based samples with dimensions of 10 × 23 mm and a thickness of 2 mm. An immittance meter (E7-14) was employed to measure electrical conductivity (σ) at low frequencies (100 Hz) using a two-contacts method [30]. For samples with a square shape (10 × 10 mm) and a thickness of 2 mm, graphite electrodes were utilized in the electrical conductivity measurements, with the experimental error not exceeding 5%.

## 3. Results and Discussion

### 3.1. Characterization of (NiZn)_1−x_Mn_x_Fe_2_O_4_/CNT Nanocomposites—XRD and TEM Studies

**Figure 1:**
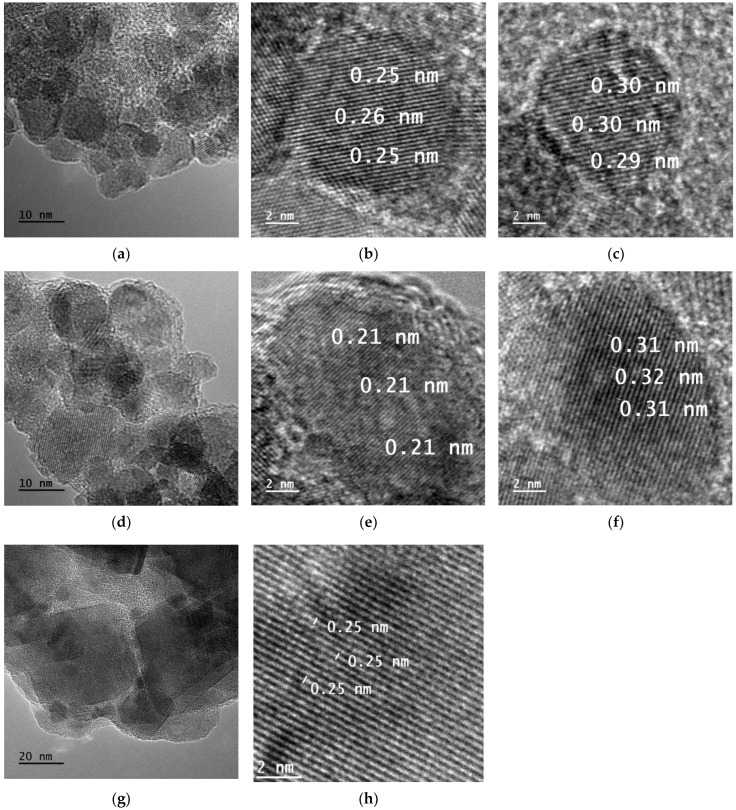
TEM images of synthesized (NiZn)_1−x_Mn_x_Fe_2_O_4_ nanoparticles: x = 0 (**a**–**c**), x = 0.5 (**d**–**f**) and x = 1 (**g**,**h**).

**Figure 2:**
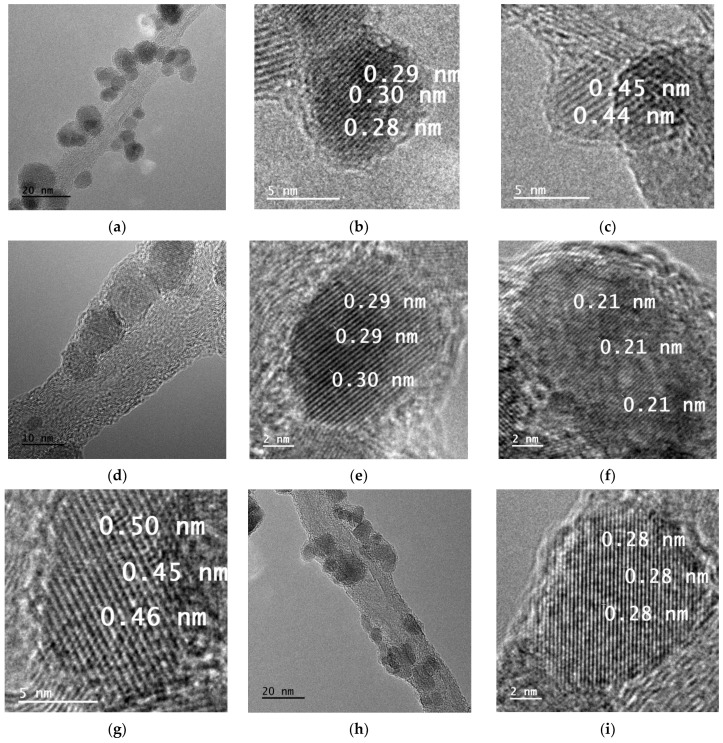
TEM images of synthesized (NiZn)_1−x_Mn_x_Fe_2_O_4_/0.05CNT nanocomposites: x = 0 (**a**–**c**), x = 0.5 (**d**–**g**) and x = 1 (**h**,**i**).

**Figure 3:**
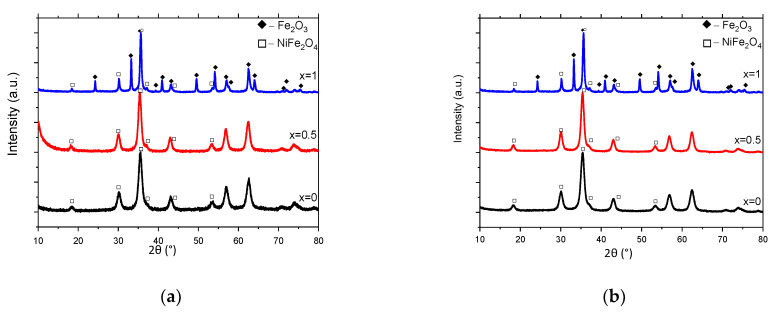
X-ray diffraction patterns of synthesized samples: (NiZn)_1−x_Mn_x_Fe_2_O_4_ (**a**) and (NiZn)_1−x_Mn_x_Fe_2_O_4_/0.05CNT (**b**).

The crystal structure and size of as-synthesized ferrites and ferrite/CNT nanocomposites are presented in Figure 1 and Figure 2, while the chemical and phase compositions studied with X-ray fluorescence and X-ray diffraction are presented in Table 1 and Figure 3, respectively. One can see that the nickel to zinc ratio in all (NiZn)_1−x_Mn_x_Fe_2_O_4_ ferrites and (NiZn)_1−x_Mn_x_Fe_2_O_4_/CNT nanocomposites is close to 1, as well as the Ni:Zn:Mn ratio, which confirms the successful synthesis of substituted ferrites.

As can be seen in TEM pictures (Figure 1 and Figure 2), both pristine ferrites and (NiZn)_1−x_Mn_x_Fe_2_O_4_/CNT nanocomposites can be characterized by nanometric structure, with a crystallite size below 20 nm. Contrary to MnFe_2_O_4_, which crystallizes mostly along the (311) direction, corresponding to an interplanar distance of 0.25–0.26 nm, the (NiZn)_1−x_Mn_x_Fe_2_O_4_ ferrites crystallize along different directions. As presented in Figure 1b, planes with a distance of 0.23–0.25 nm correspond to the (222) direction, while planes presented in Figure 1e with a distance of 0.22 nm can be assigned to the (400) direction. Illustrated in Figure 1c,f are planes with distances of 0.29–0.32 nm and with the presence of some lattice strain or crystal imperfections, corresponding most likely to the (220) direction. In the case of (NiZn)_1−x_Mn_x_Fe_2_O_4_/CNT nanocomposites, three crystallization directions have been recognized: (311), with a distance of 0.25–0.26 nm (Figure 2g); (220), with a distance of 0.30–0.34 (Figure 2e); and (111), with a distance of 0.44–0.48 (Figure 2b,c,f).

It should be mentioned that, according to the ICDD# 00-054-0964 card, the model distance for the (400) plane is 0.208 nm, while for the (222), (311), (220), and (111) planes the distances are 0.241 nm, 0.251 nm, 0.295 nm and 0.481 nm, respectively. Such a big variation from model data might be a result of lattice strain or crystal imperfections, especially due to the large number of defects in the nanometric structures.

The results of TEM and XRF techniques stand in good agreement with XRD data and confirm crystallite sizes below 15 nm for x = 0 and x = 0.5, while for x = 1, this parameter is in the range of 19 to 22 nm. It is worth mentioning that usually linear defects, such as dislocations, occur simultaneously with point defects, i.e., Frenkel defects, and both strongly affect lattice strain and crystal imperfections. The calculated dislocation densities of ferrites, as a representation of crystal imperfections, are 8.26 × 10^15^/m^2^, 6.94 × 10^15^/m^2^, and 2.77 × 10^15^/m^2^ for x = 0, x = 0.5, and x = 1, respectively. The dislocation densities for (NiZn)_1−x_Mn_x_Fe_2_O_4_/CNT nanocomposites are 6.94 × 10^15^/m^2^, 5.10 × 10^15^/m^2^, and 2.07 × 10^15^/m^2^ for x = 0, x = 0.5, and x = 1, respectively. This result shows that with an increase in manganese content, the crystal imperfections decrease as a result of crystallite growth.

Rietveld refinement analyses also show a minor increase in lattice parameters with a decrease in manganese content. For MnFe_2_O_4_, (NiZn)_0.5_Mn_0.5_Fe_2_O_4_, and (NiZn)Fe_2_O_4_ ferrites, the lattice parameters of the cubic Fd-3m structure are a = 8.400, a = 8.410, and a = 8.416, respectively. For corresponding ferrites anchored on carbon nanotubes, the lattice parameters are a = 8.406, a = 8.412, and a = 8.416, respectively. Additionally, a minor amount (about 18%) of rhombohedral Fe_2_O_3_ is present in the MnFe_2_O_4_ and MnFe_2_O_4_/CNT systems, with a size of about 40 nm. It is known that substituted NiZnMn ferrites exhibit good magnetic properties, particularly high values of magnetization [31,32]. The antiferromagnetic hematite (Fe_2_O_3_) shows a small spontaneous magnetization in the temperature range of 246–950 K, compared with substituted ferrites [33,34]. It is worth noting that pure Fe_2_O_3_ has a low attenuation ability for EM radiation [35]. Therefore, we do not expect a significant influence of this phase on the absorption properties of (NiZn)_1−x_Mn_x_Fe_2_O_4_/CNT nanocomposites as a whole and this nanocomposite was used without additional purification.

### 3.2. Electrophysical Properties of Polymer Composites Based on (NiZn)_1−x_Mn_x_Fe_2_O_4_/CNT

To create a material capable of absorbing electromagnetic radiation, achieving optimal impedance matching is crucial, and this is determined by specific material parameters, including the relative complex dielectric constant and magnetic permeability. Table 2 presents a summary of the real and imaginary components of complex permeability, complex permittivity at 9 GHz, and conductivity at low frequency (100 Hz) for both the synthesized (NiZn)_1−x_Mn_x_Fe_2_O_4_ ferrites and NiZn)_1−x_Mn_x_Fe_2_O_4_/CNT nanocomposites. It is evident that the values of ε′ and ε″ increase with the increasing concentration of carbon nanotubes in (NiZn)_1−x_Mn_x_Fe_2_O_4_/CNT nanocomposites for all x values. This results from the natural ferromagnetic resonance [36] and the heightened surface defectiveness of magnetic nanoparticles. The increased area of interactions between the magnetic nanoparticles and the conductive component of CNTs contributes to this phenomenon. The conductivity of (NiZn)_1−x_Mn_x_Fe_2_O_4_/CNT nanocomposites at a frequency of 100 Hz is approximately 2–3 orders of magnitude greater than that of (NiZn)_1−x_Mn_x_Fe_2_O_4_ ferrites.

### 3.3. Effect of Distribution of Ferrites/CNT Nanocomposites in an Epoxy Matrix on the Electromagnetic Wave Absorption Performance of (NiZn)_1−x_Mn_x_Fe_2_O_4_/CNT–ER Systems

**Figure 4:**
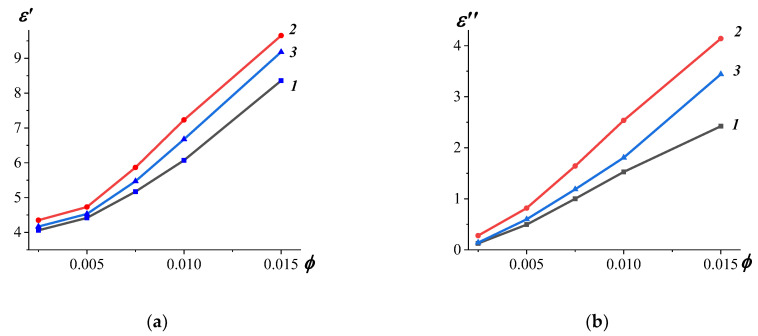
Dependencies of ε′ (**a**) and ε″ (**b**) at 9 GHz on the CNT volume fractions in the polymer composite systems (NiZn)_1−x_Mn_x_Fe_2_O_4_/0.07CNT–ER: x = 0 (*1*), x = 0.5 (*2*) and x = 1 (*3*).

Figure 4 illustrates the experimental results of measurements of the complex permittivity at 9 GHz for the systems (NiZn)_1−x_Mn_x_Fe_2_O_4_/0.07CNT (x = 0, 0.5, and 1) with the epoxy matrix. The incorporation of (NiZn)_1−x_Mn_x_Fe_2_O_4_/0.07CN nanocomposites into the epoxy resin results in an elevation of both ε′ and ε″ for all examined amounts of filler. This effect can be explained by the existence of a polarisation mechanism—interfacial polarisation in multicomponent heterogeneous media. Namely, this effect occurs in heterojunction structures due to the accumulation of charges at the conductor/insulator interfaces. In our case, when forming polymer-filled composites based on epoxy resin, a boundary layer of polymer macromolecules with reduced mobility is formed on the surface of the filler [37], which hinders the relaxation processes and contributes to the formation of a dipole layer, which causes the occurrence of interfacial polarization With increasing filler concentration in polymer composites, an increase in the interfacial specific surface area on which the interaction of components occurs. As the interfacial surface increases, the contribution of interfacial polarization increases, thereby increasing the values of ε′. As the content of carbon nanotubes modified by substituted ferrite rises in the epoxy matrix a greater number conductive paths are formed, which leads to an increase in conduction losses and an increase in ε″ values (Figure 4b). A nonlinear increase of ε′ and ε″ values in these polymer nanocomposites with subsequent increase volume content of carbon nanotubes was foumd which is associated with the presence of a percolation transition (approximately 0.005–0.015 vol. fractions) and corresponds to its threshold. It was found that the system (NiZn)_1−x_Mn_x_Fe_2_O_4_/0.07CNT–ER (x = 0.5) is characterized by the highest values of ε′ and ε″. The maximum values of the complex dielectric constant were confirmed for the (NiZn)_1−x_Mn_x_Fe_2_O_4_/CNT–ER system, which is probably due to a change in the structure of ferrite clusters on the surface of carbon nanotubes in (NiZn)_1−x_Mn_x_Fe_2_O_4_/CNT nanocomposites.

EMI shielding effectiveness (*SE*) can be quantitatively expressed using the following equation [38]:

(2) $$SE=10lg(\frac{P_{I}}{P_{t}})$$

where *P_I_* and *P_t_* are the power of the incident wave and the wave that travels through the material in decibels (dB), respectively.

In the theory of EMI shielding, as an electromagnetic wave interacts with the shielding material, the incident power undergoes division into reflected, absorbed, and transmitted power. The corresponding power coefficients of absorbance (*A*), reflectance (*R*), and transmittance (*T*) satisfy the condition *A* + *R* + *T* = 1 [39].

In the microwave absorption model [40], the transmission waves are always negligible and reflection loss (*RL*) is the difference between the initial incident wave and the final reflected wave, including all the back-propagating EM waves reflected on different surfaces and interfaces. *RL* can be calculated using the measured complex permittivity and complex permeability data for the given frequency and absorber thickness using the following equations [38,39]:

(3) $$RL\left(dB\right)=20log\left|\frac{Z_{in}-Z_{0}}{Z_{in}+Z_{0}}\right|$$

where *Z_in_* is the input impedance of absorbent and *Z*_0_ is the intrinsic impedance in air; and

(4) $$Z_{in}=Z_{0}\sqrt{\frac{\mu_{r}}{\varepsilon_{r}}}tanh(j\frac{2\pi fd}{c}\sqrt{\mu_{r}\varepsilon_{r}})$$

where $\mu_{r}=\mu^{\prime }-ί\mu^{\prime \prime }$ is the relative complex magnetic permeability ($\mu^{\prime }$, $\mu^{\prime \prime }$—real and imaginary components of the complex permeability, respectively), $\varepsilon_{r}=\varepsilon^{\prime }-ί\varepsilon^{\prime \prime }$ is the relative complex dielectric permittivity ($\varepsilon^{׳}$, $\varepsilon^{״}$—real and imaginary components of the complex permittivity, respectively), *f* is the electromagnetic wave frequency, *d* is the thickness of sample, and *c* is the velocity of light in a vacuum.

**Figure 5:**
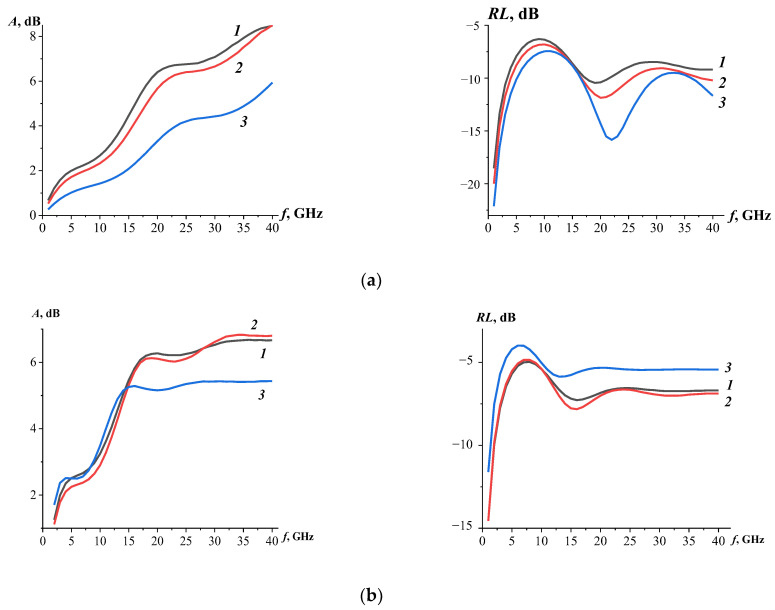
Frequency dependencies of EM wave absorption coefficients (left column) and reflection losses (right column) of (NiZn)_1−x_Mn_x_Fe_2_O_4_ substituted ferrites (**a**) and (NiZn)_1−x_Mn_x_Fe_2_O_4_/0.07CNT nanocomposites (**b**): x = 0 (*1*), x = 0.5 (*2*) and x = 1 (*3*). The thickness of the samples was 4 mm.

The results presented in Figure 5a demonstrate the change in absorption in the whole frequency range at different manganese ion contents in (NiZn)_1−x_Mn_x_Fe_2_O_4_ ferrite at x = 0, 0.5, and 1. A significant increase in the bandwidth (*RL*) and its broadening at 9 GHz has been observed; namely, for samples at x = 0 and x = 1, the bandwidth corresponds to 4.4 GHz and 14 GHz, respectively. At the same time, a change in reflection loss from −10.5 dB to −15.8 dB was observed for the (NiZn)_1−x_Mn_x_Fe_2_O_4_ substituted ferrite samples.

The EM wave absorption processes in nanocomposites based on carbon nanotubes decorated with substituted ferrites (Figure 5b) are more efficient in the frequency range of 12 GHz to 20 GHz, most likely as a result of better impedance matching characteristics than for unmodified ferrite samples. Meanwhile, the minimum values of *RL* shifted to the low frequency region and the values also decreased due to an increase in the conductivity of the composites. Analyzing the mechanisms of electromagnetic wave absorption shows that the increase in losses, associated to both eddy currents and conduction losses, is a result of increased conductivity [41]. However, in the case of high conductivity, the impedance of the material is relatively small compared to the impedance of air and, as a result, almost all of the electromagnetic waves will be reflected. The loss of energy of the incident EM wave by absorbing materials is realized due to the joint action of the absorption and interference mechanism. In this case, the absorption mechanism is mainly determined by the impedance matching and the ability to attenuate the EM wave depending on the ratio of dielectric losses and magnetic losses. The penetration of the EM wave into the absorbing material depends on the degree of the impedance matching between the surface of the CNT-based material modified with substituted ferrites and the free space. The use of polymer matrices transmitting EM waves allows to reduce the effect of reflection of microwaves from the surface of the absorbing material. In our case, epoxy resin is a material that transmits EM waves and performs the impedance matching between (NiZn)_1−x_Mn_x_Fe_2_O_4_/0.07CNT nanocomposite and the free space.

**Figure 6:**
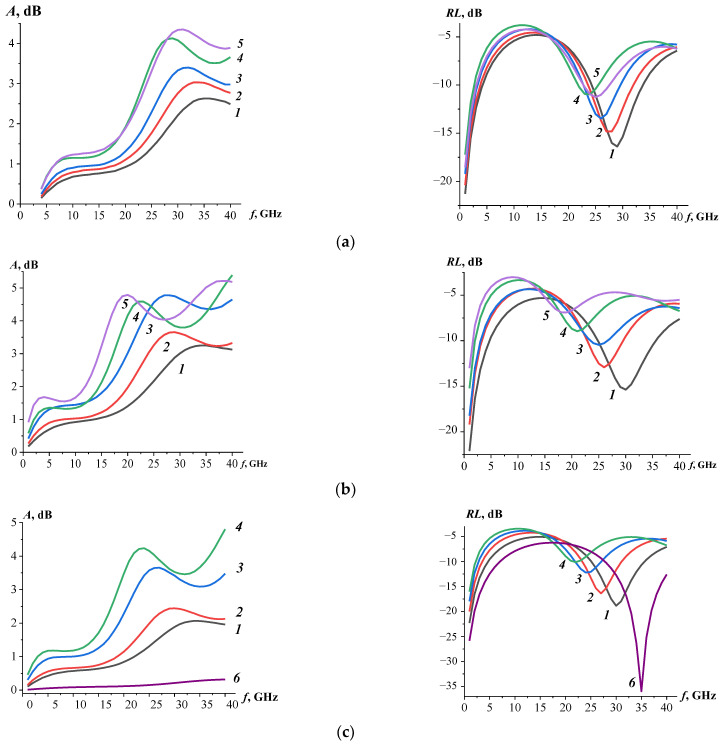
Frequency dependencies of EM wave absorption coefficients (left column) and reflection losses (right column) in polymer-based systems (NiZn)_1−x_Mn_x_Fe_2_O_4_/0.07CNT–ER: x = 0 (**a**), x = 0.5 (**b**), and x = 1 (**c**). Volume fractions of CNT: *1*—0.0025; *2*—0.005; *3*—0.0075; *4*—0.01; *5*—0.015; *6*—0 (pure ER). The thickness of the samples was 2.5 mm.

Figure 6 shows the calculated absorption and reflection loss curves of polymer systems based on an epoxy matrix and carbon nanotubes modified with substituted ferrites. For comparative analysis of the absorption and reflection processes polymer systems depending on the filler content, a fixed thickness was chosen. Since the interference minima will shift in frequency as the sample thickness changes [42,43], while the absorption at the corresponding sample thickness will not change significantly. As the filler volume content increases in the epoxy matrix for (NiZn)_1−x_Mn_x_Fe_2_O_4_/0.07CNT–ER systems with a doping amount of manganese ions (x = 0, 0.5, and 1), there is an observed increase in the maximum peak values of the absorption coefficient, accompanied by a shift of these maxima to a lower frequency region.

In particular, the (NiZn)_1−x_Mn_x_Fe_2_O_4_/0.07CNT–ER system is characterized by shifts in the maximum peak value of the absorption coefficient as the filler content increases. For x = 0, there is a shift from 35.2 GHz to 30.4 GHz; for x = 0.5, a shift from 33.8 GHz to 19.7 GHz; and for x = 1, a shift from 34.2 GHz to 23.3 GHz. Additionally, for these polymer systems (x = 0, 0.5, and 1), the minimum reflection loss peaks and their shift to the low-frequency region are also observed. Specifically, the samples based on (NiZn)_1−x_Mn_x_Fe_2_O_4_/0.07CNT (x = 1) with 0.0025 vol. % demonstrated electromagnetic absorption performance at frequencies around 30 GHz with *RL* < −18.7 dB. It is noteworthy that, for all polymer composites with a filler content of 0.0025 volumetric fraction, the highest bandwidth (*RL* < −10 dB) was determined. The polymer system based on (NiZn)_1−x_Mn_x_Fe_2_O_4_/0.07CNT at x = 0.5 exhibited the highest value of the bandwidth (*RL* < −10 dB) at 10.8 GHz. With the increase in the concentration of carbon nanotubes in polymer systems, a narrowing of the bandwidth and a decrease in the reflection loss were observed. It is known that dielectric losses, magnetic losses, and impedance matching characteristics affect the absorption properties of materials. The increasing content of carbon nanotubes in polymer-based systems, characterized by a high aspect ratio, results in the formation of a conductive network and leads to an increase in dielectric losses. However, the increase in the complex dielectric permittivity of polymer systems (Figure 4) affects impedance matching and results in more significant reflection.

In this study, a comparative analysis of the absorption properties at hight high-frequency region of various polymer composites based on epoxy resin and various fillers published in recent years was conducted (Table 3). It is noteworthy that the bandwidth of RL, a crucial factor for potential applications, was found to be one of the highest in (NiZn)_1−x_Mn_x_Fe_2_O_4_/CNT–ER. These results confirm that this type of composite is a very promising material for use as an electromagnetic wave-absorbing coating.

## 4. Conclusions

Nano-sized (NiZn)_1−x_Mn_x_Fe_2_O_4_ ferrites (x = 0, 0.5, and 1) and (NiZn)_1−x_Mn_x_Fe_2_O_4_/CNT nanocomposites were synthesized through a one-pot co-precipitation process. The concentration of carbon nanotubes (CNTs) in the (NiZn)_1−x_Mn_x_Fe_2_O_4_/CNT system ranged from 0.05 to 0.07 vol. fractions, and the particle size of substituted ferrites on the surface of carbon nanotubes was within the range of 5–20 nm.

An increase in the concentration of CNTs in (NiZn)_1−x_Mn_x_Fe_2_O_4_/CNT composites led to elevated values of ε′ and ε″ for all values of x. The (NiZn)_1−x_Mn_x_Fe_2_O_4_/CNT nanocomposites exhibited conductivity at a frequency of 100 Hz approximately 2–3 orders of magnitude higher than that observed for (NiZn)_1−x_Mn_x_Fe_2_O_4_ ferrites. In the frequency range of 12 GHz to 20 GHz, the electromagnetic wave absorption processes in nanocomposites based on carbon nanotubes decorated with substituted ferrites were more efficient, demonstrating good impedance matching characteristics. Additionally, the absorption coefficient of electromagnetic waves in the frequency range of 15–25 GHz for (NiZn)_1−x_Mn_x_Fe_2_O_4_/0.07CNT (x = 0, 0.5, and 1) nanocomposites was up to 1.5 times higher than that for unmodified substituted ferrites.

Polymer-based systems incorporating (NiZn)_1−x_Mn_x_Fe_2_O_4_/CNT nanostructures were constructed using amorphous epoxy resin as a matrix. As the filler volume content increased in the epoxy matrix for (NiZn)_1−x_Mn_x_Fe_2_O_4_/0.07CNT–ER systems with different ion doping (x = 0, 0.5, and 1), there was a rise in the maximum peak values of the absorption coefficient, and these maxima shifted toward lower frequency region. All polymer composites with a filler content of 0.0025 volumetric fraction demonstrated the highest bandwidth (*RL* < −10 dB). The optimal range of (NiZn)_1−x_Mn_x_Fe_2_O_4_/0.07CNT filler concentrations, corresponding to the absorption bandwidth with reflection loss below −10 dB for polymer composites, was found to be from 0.005 to 0.0075 vol. fractions. The microwave adsorption properties of (NiZn)_1−x_Mn_x_Fe_2_O_4_/0.07CNT–ER (x = 0.5) systems indicated that the maximum absorption bandwidth with a reflection loss below −10 dB is about 11 GHz.

This study successfully demonstrated that the incorporation of carbon nanotubes decorated with substituted ferrite nanoparticles into an epoxy matrix allows for the regulation of absorbing properties in the microwave region at specific studied filler concentrations.

## Figures and Tables

**Table 1:** The chemical composition of synthesized (NiZn)_1−x_Mn_x_Fe_2_O_4_ ferrites and (NiZn)_1−x_Mn_x_Fe_2_O_4_/CNT hybrids determined using X-ray fluorescence.

Sample	Weight Amount, %				Ni:Zn	Ni:Zn:Mn	Fe:(Ni,Zn,Mn)
	Fe	Ni	Zn	Mn			
(NiZn)_1−x_Mn_x_Fe_2_O_4_ (x = 0)	68.36	15.30	16.34	0.00	0.94	0.00	2.16
(NiZn)_1−x_Mn_x_Fe_2_O_4_ (x = 0.5)	72.20	9.17	10.36	8.27	0.89	1.18	2.60
NiZn)_1−x_Mn_x_Fe_2_O_4_/0.07CNT (x = 0)	68.23	15.30	16.48	0.00	0.93	0.00	2.15
(NiZn)_1−x_Mn_x_Fe_2_O_4_/0.07CNT (x = 0.5)	75.04	8.37	9.15	7.44	0.91	1.18	3.01

**Table 2:** The real and imaginary part of complex permeability and complex permittivity at 9 GHz, and the conductivity of spinel ferrites (NiZn)_1−x_Mn_x_Fe_2_O_4_ and their nanocomposites with CNTs.

Sample	x	μ′	μ″	ε′	ε″	σ, (Om∙cm)^−1^ (100 Hz)
(NiZn)_1−x_Mn_x_Fe_2_O_4_	0	1.08	0.04	3.1	1.7	2 × 10^−5^
	0.5	1.02	0.02	2.5	1.4	3 × 10^−5^
	1	1.07	0.01	2.6	0.7	5 × 10^−5^
(NiZn)_1−x_Mn_x_Fe_2_O_4_/0.05CNT	0	1.06	0.02	2.8	2.2	4 × 10^−3^
	0.5	1.04	0.04	3.1	2.1	6 × 10^−3^
	1	1.02	0.02	5.0	5.3	2 × 10^−2^
(NiZn)_1−x_Mn_x_Fe_2_O_4_/0.07CNT	0	1.08	0.02	3.6	2.4	1 × 10^−2^
	0.5	1.06	0.06	3.9	3.3	2 × 10^−2^
	1	1.08	0.04	5.7	5.8	7 × 10^−2^

**Table 3:** The microwave absorption properties of polymer composite materials reported in literature compared with experimental data.

Absorber/Matrix	Minimum *RL* (dB)	Frequency of Minimum *RL* (GHz)	Sample Thickness d (mm)	Bandwidth of *RL* < −10 dB (GHz)	Ref.
Ni_0.6_Zn_0.4_Fe_2_O_4_/epoxy resin	−37.47	12.60	3.1	9.00	[44]
CNT (2.5 wt. %)—epoxy resin	−17.00	9.30	2.0	2.10	[45]
CoFe_2_O_4_—MWCNT (2 wt. %)—epoxy resin	−28.00	9.00	3.0	4.00	[46]
SrFe_7.6_Co_1.2_Ti_1.2_O_19_/epoxy resin	−42.00	6.30	4.0	4.00	[20]
EG (44.7 vol. %)—epoxy resin	−23.83	8.40	4.0	10.8	[47]
Ni_0.5_Zn_0.5_Fe_2_O_4_/0.07CNT–ER (CNT = 0.0025 vol. %)	−16.40	28.93	2.5	9.46	This work
Ni_0.25_Zn_0.25_Mn_0.5_Fe_2_O_4_/0.07CNT–ER (CNT = 0.0025 vol. %)	−15.41	29.96	2.5	10.55	This work
MnFe_2_O_4_/0.07CNT–ER (CNT = 0.0025 vol. %)	−18.8	30.01	2.5	10.62	This work

## Data Availability

Data are contained within the article.
